# Evaluation of cardiothoracic ratio as a potential predictor of cardiovascular abnormalities in individuals with type II diabetes mellitus: a case-control study

**DOI:** 10.25122/jml-2024-0029

**Published:** 2024-07

**Authors:** Mohammed Abuelnor, Asmaa Sharif, Bassam Farhan Alakhras, Khaled Alattar, Muruj Shehab, Ashwaq Alfayez, Fatimah Ahmorawdh, Souhayla Almasri, Reeouf Aldossry, Ghunyah Alfaraj

**Affiliations:** 1 Department of Basic Medical Science, College of Medicine, Dar Al Uloom University, Riyadh, Saudi Arabia; 2 Department of Forensic Medicine and Clinical Toxicology, Faculty of Medicine, Tanta University, Tanta, Egypt; 3 Department of Clinical Medical Science, College of Medicine, Dar Al Uloom University, Riyadh, Saudi Arabia; 4 Department of Internal Medicine, Prince Mohammed bin Abdulaziz Hospital, Riyadh, Saudi Arabia; 5 College of Medicine, Dar Al Uloom University, Riyadh, Saudi Arabia

**Keywords:** cardiothoracic ratio, diabetes mellitus, cardiac disorders

## Abstract

Cardiovascular complications represent a significant health concern for individuals with diabetes mellitus. The relationship between diabetes and cardiovascular diseases is complex and multifaceted, involving a variety of pathophysiological mechanisms. This study aimed to investigate the potential role of the cardiothoracic ratio as a prognostic tool for cardiovascular disorders in patients with diabetes. A retrospective case-control study of 530 adult patients referred to a tertiary care hospital in Saudi Arabia was conducted. Medical records, including chest X-rays, were analyzed to determine the cardiothoracic ratio. Patients diagnosed with diabetes who experienced cardiac disorders had significantly higher cardiothoracic ratios compared to patients with diabetes alone and controls. HbA1c was significantly elevated among patients with diabetes and cardiovascular disorders (mean = 71.5 ± 25.43 mmol/mol) compared to the other patients. There was a significant positive correlation between the duration of diabetes and the cardiothoracic ratio (r = 0.64, *P* < 0.001). Furthermore, the cardiothoracic ratio above 0.51 was a good discriminator of cardiovascular disorders in patients with diabetes, with an area under the curve of 0.737, sensitivity of 97.1%, and specificity of 87.2%. This study provided comprehensive evidence supporting the association between cardiothoracic ratio and subsequent cardiovascular adverse outcomes in patients with diabetes. We recommend adopting the cardiothoracic ratio as a valuable prognostic tool for risk stratification among people with diabetes.

## INTRODUCTION

Diabetes mellitus (DM) is a widespread metabolic disorder characterized by chronic elevation of blood glucose levels, which poses significant health risks globally [1]. Cardiovascular complications represent a major health concern for individuals with DM [2], representing a leading cause of morbidity and mortality. However, the relationship between DM and cardiovascular diseases is complex and multifaceted, involving a variety of pathophysiological mechanisms [3]. The pathophysiology of cardiovascular complications in diabetes involves hyperglycemia-induced oxidative stress, endothelial dysfunction, inflammation, dyslipidemia, insulin resistance, and the interplay between microvascular and macrovascular complications [4]. Understanding these mechanisms is crucial in preventing the risk of developing atherosclerosis, myocardial infarction, heart failure, and other cardiovascular disorders [5]. Therefore, early detection and risk stratification are crucial for optimal management and preventing cardiac complications in patients with diabetes [6].

Cardiothoracic ratio (CTR) is a radiological measurement obtained from a simple chest X-ray, which quantifies the ratio of the maximum diameter of the heart to the maximum diameter of the chest cavity [7]. An elevated CTR often indicates cardiac enlargement, a common feature in patients with heart failure [8]. Apart from heart failure, cardiac enlargement results from various conditions, including cardiomyopathy and valvular diseases [9]. One of the most studied aspects of CTR is its role in predicting heart failure outcomes. Several studies have demonstrated a significant association between an increased CTR and a higher risk of heart failure-related hospitalizations and mortality [10]. Though several studies have suggested that cardiac enlargement is associated with long-lasting diabetes, the precise association between the severity of diabetes and the extent of cardiac enlargement is not fully understood [11,12].

In the current study, we investigated the prognostic role of CTR as a non-invasive, cost-effective screening tool for early detection of impending cardiovascular complications among patients with diabetes. This study aimed to comprehensively evaluate the association between CTR measurements and subsequent cardiovascular adverse outcomes in patients with diabetes, including the development of heart failure, coronary artery disease, valvular heart disease, and cardiomyopathy. The present work sought to provide a deeper understanding of how CTR can serve as a valuable prognostic tool in the context of DM to enhance risk stratification, guide clinical management decisions, and improve the long-term cardiovascular prognosis for patients with diabetes.

## MATERIAL AND METHODS

### Study design, setting, and sample size

This retrospective case-control study utilized archived data from adult patients admitted to Prince Muhammed Bin Abdul-Aziz Hospital, Riyadh, Kingdom of Saudi Arabia, between 2019 and 2023. The retrieved data included only patients who underwent chest X-rays (CXRs).

Convenience sampling was adopted to approach the largest number of patients who met the inclusion criteria. The literature review indicated a wide range of expected frequencies for cardiovascular complications among patients with diabetes [13-15]. According to Kengne *et al*. [16], about 50% of patients with diabetes developed at least one cardiovascular complication. Based on this, the minimum sample size required to meet the study objectives was estimated to be at least 370. A margin of error of ± 5.094% was adopted with a 95% confidence level. An additional 160 controls were included to ensure robust statistical power, resulting in a total sample size of 530 cases. Sample size calculations were performed using Open-Source Epidemiologic Statistics for Public Health software, Version 3.01.

### Patient grouping and outcomes

Enrolled patients were classified into three distinct groups. Group I was the control group comprising 160 individuals without known cardiac or diabetes disorders or risk factors who attended the hospital and conducted CXRs for reasons not related to the current study. Group II included 247 patients diagnosed with DM without cardiovascular diseases. Group III consisted of 123 patients diagnosed with DM and one or more of the following cardiovascular diseases, including the development of heart failure, coronary artery disease, valvular heart disease, and cardiomyopathy. Cases within each group were carefully matched based on sex and age to ensure comparability across groups.

### Inclusion criteria

Adult patients aged 18 years and older admitted to Prince Muhammed Bin Abdul-Aziz Hospital during the study period and had a confirmed diagnosis of type 2 diabetes mellitus (T2DM) for at least one year were eligible for inclusion in Groups II and III. Only patients with complete medical records containing high-quality CXRs were included. For the control group (Group I), adult individuals who underwent CXRs unrelated to the study but had no cardiovascular, respiratory, or mediastinal disorders that could affect the cardiothoracic ratio (CTR) were considered eligible.

### Exclusion criteria

Patients diagnosed with type I DM, congenital heart diseases, or infectious heart diseases, and patients with a history of prior cardiac surgery or cardiac catheterization were excluded. Also, patients with incomplete medical records or CXRs with discrepancies in CTR measurements were excluded from the study cohort.

### Data collection tool

Demographic information, including age and sex, was recorded for each participant. The body mass index (BMI) and glycated hemoglobin A1c (HbA1c) were also reported. HbA1c is a relevant laboratory parameter that provides a reliable assessment of long-term blood sugar control [17].

**Figure 1:**
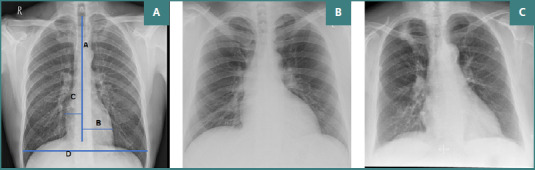
Chest radiographs and cardiothoracic ratio measurements. A, Chest radiograph demonstrating measurements for calculating the cardiothoracic ratio (CTR) in normal size heart of DM patient; B, Chest radiograph of moderate cardiomegaly in DM patient; C, Chest radiograph of severe cardiomegaly in DM patient.

The cardiothoracic ratio (CTR) was measured by dividing the cardiac diameter, the sum of the lengths from the midline to both the right and left heart borders, by the thoracic diameter, the internal chest width, on posteroanterior chest X-rays. This measurement was performed using Micro DICOM Software 2020 - 3.7.7 x 86 version, as illustrated in Figure 1 (A-C). Values up to 0.50 were considered within the normal range for CTR. Two investigators independently assessed all measurements at different time points. The inter-rater and intra-rater errors were computed. Only images with negligible variations were considered for analysis.

### Statistical analysis

Statistical analysis was performed using the Statistical Package for the Social Sciences (SPSS) Software Package version 26. The data was presented as means and standard deviation, or numbers and percentages. T-test was used to compare patients with and without diabetes, and ANOVA was used to compare the three studied groups. The chi-square test was conducted to examine the relationship between cardiomegaly and angiopathy, as well as their association with cardiovascular disorders in individuals diagnosed with DM. Pearson’s correlations assessed the relationships between various parameters. Following this analysis, Receiver Operating Characteristic (ROC) curves were generated to evaluate the accuracy of CTR as a predictor of cardiovascular disorders. Statistical significance was set at *P* values less than 0.05, and results were reported with a 95% confidence interval (CI).

## RESULTS

**Figure 2:**
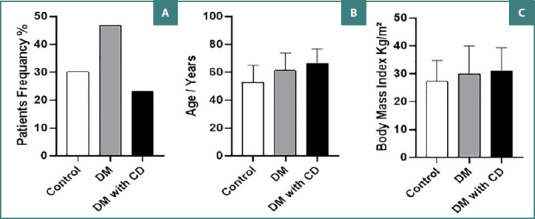
Demographic and clinical characteristics of study participants. A, Distribution of patient frequency by group (Control, DM, DM with cardiac disorders); B, Average age of patients in each group. C, Average BMI of patients in each group.

The present study included 530 patients, among whom 285 were men (53.77%) and 245 were women (46.23%). The control group consisted of 160 patients (30.19%) with an average age of approximately 52.6 ± 12.5 years, ranging from 30 to 76 years. Patients with diabetes constituted 69.91% of the total cases, with an average age of 61.27 ± 11.73 years, ranging from 31 to 71 years. Within this group, 247 patients (47.60%) had diabetes without cardiovascular diseases, with an average age of approximately 63 ± 12.09 years (ranging from 31 to 71 years). The remaining 123 patients (55.56%) had both diabetes and cardiovascular diseases, with an average age of 60.66 ± 11.41 years, ranging from 25 to 55 years (Figure 2A).

As shown in Figure 2B, controls were generally younger than patients with diabetes (52.60 ± 12.16 years). However, no significant age differences existed between the control group and Groups II and III (*P* > 0.05). BMI measurements across the groups revealed median values of 27.32 ± 8.51, 30.06 ± 11.30, and 31.16 ± 9.21 Kg/m^2^ for Groups I, II, and III, respectively. However, these differences did not reach statistical significance, as demonstrated in Figure 2C.

**Figure 3:**
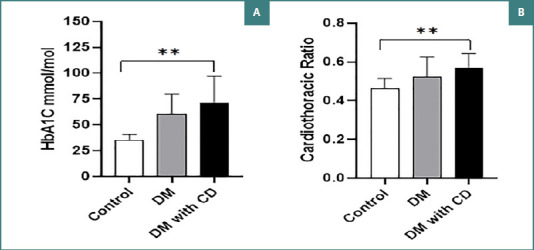
Glycemic control and cardiothoracic ratio in study groups. A, HbA1C levels across different groups. B, CTR across different groups.

The level of HbA1c was significantly lower among the control group, where it measured 34.81 ± 5.99 mmol/mol, indicating normal blood glucose level over the last three months, compared to Group II (60.57 ± 18.96 mmol/mol), and Group III (71.5 ± 25.43 mmol/mol). The variations in HbA1c among the participants were statistically significant (*P* < 0.001), as shown in Figure 3A. Furthermore, patients in Group III had the highest CTR (0.56 ± 0.07) compared to Group II (0.52 ± 0.09) and Group I, which showed the lowest level of CTR (0.46 ± 0.02) (Figure 3B). Significant variations were noticed among the groups (*P* < 0.001), indicating larger cardiac size in patients diagnosed with DM. However, the correlation between CTR and HbA1c was not significant (*P* > 0.05).

**Figure 4:**
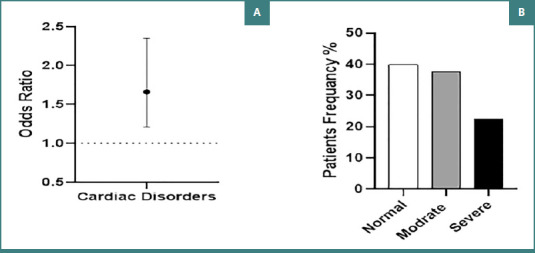
Analysis of cardiac disorders and cardiothoracic ratio. A, Odds ratio for the presence of cardiac disorders in patients with diabetes compared to control patients; B, Frequency distribution of cardiomegaly severity (normal, moderate, severe) among patients with diabetes.

Figure 4A illustrates that patients with DM had 1.66 times higher odds of cardiac enlargement compared to the control group (95% CI, 2.35–1.21). Furthermore, 147 patients (39.86%) had a CTR within the normal range (≤0.50), while 140 patients (37.86%) were classified as having a moderate stage of cardiomegaly (CTR ranged between 0.50 and 0.60). Additionally, 83 patients (22.46 %) diagnosed with DM were categorized with severe cardiomegaly (CTR ≥ 0.60), as shown in Figure 4B.

Regarding the association between cardiomegaly and angiopathy, 132 participants without angiopathy had no evidence of cardiomegaly, whereas 57 with angiopathy showed manifestations of cardiomegaly. Among those with angiopathy, 163 patients had cardiomegaly, while only 18 did not. A significant association between cardiomegaly and angiopathy was noticed (*χ*^2^ = 137.604, *P* < 0.001) as shown in Table 1. Furthermore, among individuals without cardiovascular disorders, 121 did not have cardiomegaly compared to 76 patients with cardiomegaly. A statistically significant association was noticed between cardiomegaly, angiopathy, and cardiovascular disorders (*χ*^2^ = 76.209, *P* < 0.001).

**Table 1:** Chi-square test showing distribution of patients with diabetes according to cardiomegaly, angiopathy, and cardiac disorders

Chi-Square Tests									
		Cardiomegaly					Angiopathy		
		Absent	Present	Total	Test of Sig.	*P*	Absent	Present	Total
Angiopathy	Absent	132	57	189	137.604	<0.001			
	Present	18	163	181					
	Total	150	220	370					
Cardiac Disorders	Absent	121	76	197	76.209	<0.001	145	52	197
	Present	29	144	173			44	129	173
	Total	150	220	370			189	181	370
Test of Sig.							85.534		
*P*							<0.001		

The results revealed significant associations between cardiomegaly and angiopathy (χ^2^ = 137.604, P < 0.001), cardiomegaly and cardiac disorders (χ^2^ = 76.209, P < 0.001), as well as angiopathy and cardiac disorders (χ^2^ = 85.534, P < 0.001).

The frequency of angiopathy in relation to cardiovascular disorders showed a significant association. Only 52 patients without angiopathy had cardiovascular disorders, while 129 patients with angiopathy had cardiovascular disorders, indicating a strong relationship between angiopathy and cardiovascular disorders (*χ*^2^ = 85.534, *P* < 0.001). These findings highlighted a significant association between cardiomegaly and angiopathy, both of which are influential in the presence or absence of cardiovascular disorders in patients with diabetes.

**Figure 5:**
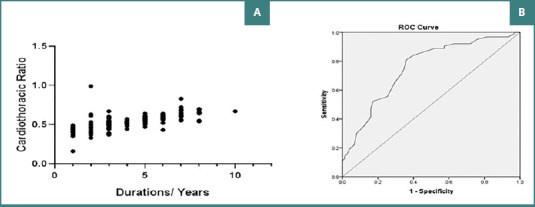
Correlation and predictive power of cardiothoracic ratio. A, Scatter plot showing the correlation between the duration of diabetes and CTR; B, ROC curve illustrating the sensitivity and specificity of CTR in predicting cardiovascular disorders in patients with diabetes.

Figure 5A demonstrates a moderate positive correlation between the duration of diabetes and CTR (r = 0.64, *P* < 0.001). Moreover, the ROC curve shown in Figure 5B shows an area under the curve (AUC) of 0.754 (95% CI, 0.673–0.835), indicating a good discrimination power of CTR in distinguishing patients with diabetes at risk of developing cardiovascular disorders from those who were not. At a cutoff of more than 0.51, the CTR could predict the incidence of cardiovascular disorders among patients with diabetes with a good performance and an overall accuracy of 73.7% (sensitivity of 97.1% and specificity of 87.2%; *P* <0.001).

## DISCUSSION

Our study delves into the intricate relationship between DM and cardiovascular disorders, with a specific focus on the utility of CTR as an early predictor of cardiovascular disorders among this category of patients. Cardiomyopathies encompass a group of heart muscle disorders that significantly affect cardiac structure and function. CTR can serve as a valuable prognostic tool in differentiating between various cardiomyopathic subtypes. For instance, dilated cardiomyopathy is often associated with an increased CTR. Accurate subtype identification based on CTR can help predict the course of the disease and guide appropriate management [18].

Coronary artery disease is a leading cause of cardiac events, including myocardial infarction. Research has explored the prognostic significance of CTR in patients with coronary artery diseases [8]. While CTR alone is not a specific marker for coronary artery disease, it can provide valuable information when combined with clinical and imaging data. In some cases, an increased CTR may be associated with extensive coronary artery disease, which could impact the prognosis and the choice of revascularization strategies [19]. CTR is particularly useful in assessing patients with valvular heart disease. An elevated CTR can indicate left ventricular dilation and hypertrophy, typical responses to chronic valvular regurgitation [20]. The extent of cardiac enlargement, as reflected by CTR, can help clinicians determine the cardiovascular illness and the need for surgical intervention. Additionally, CTR can be used as a follow-up tool to monitor changes in cardiac size post-surgery, aiding in prognosis assessment [21].

Furthermore, and in agreement with the current study, enlarged CTR was described as an indicator of impending cardiovascular impairment. It was elicited that an increased CTR in the presence of pulmonary hypertension suggested right-sided heart failure, indicating a poor prognosis. Monitoring CTR in these patients helped assess the response to treatment and predict outcomes [22].

The current study revealed that patients with diabetes mellitus who also had cardiovascular disorders exhibited significantly higher HbA1c levels compared to those with DM alone and to the control group. These findings were consistent with previous studies indicating an association between poor glycemic control and increased cardiovascular risk [23]. This reaffirms the importance of stringent glycemic management in individuals with DM and underscores the need for targeted interventions to mitigate cardiovascular complications.

Similar to the current study, which proved significantly higher CTR in patients with DM and cardiovascular diseases compared to those with DM only and healthy controls, previous research has also shown larger cardiac size in patients with DM. Likewise, CTR categorization revealed that severe cardiomegaly was present in patients diagnosed with DM [24]. This further supports the findings of previous studies that reported an increased prevalence of cardiomegaly in individuals with DM [25]. The present study demonstrated that individuals with DM had higher odds of cardiac enlargement compared to controls. This finding is consistent with previous literature suggesting an association between T2DM and cardiac enlargement [26]. This is consistent with the well-documented pathophysiological changes in the heart associated with DM [27]. These findings further substantiate the increased likelihood of cardiac enlargement in patients with DM, emphasizing the heightened cardiovascular risk in the studied cohort.

The present work highlighted the significant interplay between cardiomegaly, angiopathy, and cardiovascular disorders, as well as angiopathy and cardiovascular disorders in patients with DM. These associations underscore the multifactorial nature of cardiovascular complications in this population, highlighting the potential synergy between structural and microvascular cardiac alterations and support and expand upon findings from previous studies [28].

Furthermore, the present study suggested that an increase in the duration of diabetes correlates with a higher likelihood of cardiac enlargement. This insight into the temporal relationship between diabetes duration and cardiac changes further informs the potential utility of CTR in tracking disease progression and corroborates previous research findings indicating a relationship between diabetes duration and cardiac changes [29]. The high area under the curve (AUC) and the excellent sensitivity and specificity values of CTR underscore its accuracy in distinguishing patients at risk of cardiovascular disorders and assessing disease severity [30]. These findings underscore the potential clinical relevance of incorporating CTR into routine assessments for this population.

### Limitations

Although the obtained findings contribute to the existing body of knowledge by incorporating CTR as a potential diagnostic tool in this vulnerable category of population, several challenges and limitations are associated with using CTR as a prognostic marker for cardiac disorders in patients with DM. The most significant challenge is the lack of a standardized CTR cutoff value that allows uniform interpretation across studies. Additionally, CTR measurements are sensitive to variations in chest shape, body habitus, and radiological techniques, making comparisons between different populations and studies challenging. Prospective studies with larger, diverse cohorts are essential to validate our results and establish CTR as a robust diagnostic tool.

## CONCLUSION

This study provides valuable insights into the complex interplay of diabetes and cardiovascular disorders, offering a potential avenue for early identification of patients with diabetes at risk of developing cardiovascular complications. CTR was proved to be a substantially useful economic tool stratifying patients with diabetes at risk of developing cardiovascular disorders. Furthermore, CTR can quantify the severity and progression of DM. Incorporating CTR into routine clinical assessments for patients with diabetes could enhance decision-making and facilitate the implementation of preventive measures. By identifying at-risk individuals earlier, CTR may reduce the likelihood of requiring surgical interventions and improve overall patient outcomes.

## Data Availability

Data supporting the findings of this study are available from the corresponding author upon request.
